# Author Correction: Highly accurate determination of heterogeneously stacked Van-der-Waals materials by optical microspectroscopy

**DOI:** 10.1038/s41598-023-28605-0

**Published:** 2023-01-25

**Authors:** Andreas Hutzler, Birk Fritsch, Christian D. Matthus, Michael P. M. Jank, Mathias Rommel

**Affiliations:** 1 Electron Devices (LEB), Friedrich-Alexander University Erlangen-Nürnberg, Cauerstraße 6, 91058 Erlangen, Germany; 2 Circuit Design and Network Theory, Technische Universität Dresden, Helmholtzstraße 18, 01069 Dresden, Germany; 3 Fraunhofer Institute for Integrated Systems and Device Technology IISB, Schottkystraße 10, 91058 Erlangen, Germany

Correction to: *Scientific Reports* https://doi.org/10.1038/s41598-020-70580-3, published online 13 August 2020

The original version of this Article contained an error in Equation 1.

1 $${W}_{GF}\left(\varphi \right)=\frac{1}{\sqrt{2\pi {\sigma }^{2}}}\mathrm{exp}\left(-\frac{1}{2}\cdot {\left(\frac{3}{N}\sqrt{\frac{{\left(2n-1\right)}^{2}{\varphi }^{2}}{2}}\right)}^{2}\right)$$

now reads:

1 $${W}_{GF}\left(\varphi \right)=\frac{1}{\sqrt{2\pi {\sigma }^{2}}}\mathrm{exp}\left(-\frac{1}{2}\cdot {\left(3\cdot \frac{2n-1}{2N}\right)}^{2}\right)$$

As a result, Equation 2 was incorrect.

2 $$R\left(\lambda \right)=\sum_{n=1}^{N}R\left(\lambda ,{\varphi }_{n}\right)\cdot {W}_{NA}\cdot {W}_{GF}=\sum_{n=1}^{N}R\left(\lambda ,{\varphi }_{n}\right)\frac{2n-1}{{N}^{2}} \frac{1}{\sqrt{2\pi {\sigma }^{2}}}\mathrm{exp}\left(-\frac{1}{2}{\left(\frac{3}{N}\sqrt{\frac{{\left(2n-1\right)}^{2}{\varphi }_{n}^{2}}{2}}\right)}^{2}\right)$$

now reads:

2 $$R\left(\lambda \right)=\sum_{n=1}^{N}R\left(\lambda ,{\varphi }_{n}\right)\cdot {W}_{NA}\cdot {W}_{GF}=\sum_{n=1}^{N}R\left(\lambda ,{\varphi }_{n}\right)\frac{2n-1}{{N}^{2}} \frac{1}{\sqrt{2\pi {\sigma }^{2}}}\mathrm{exp}\left(-\frac{1}{2}\cdot {\left(3\cdot \frac{2n-1}{2N}\right)}^{2}\right)$$

The original Article has been corrected.

